# Dissociative and analgesic properties of ketamine are independent and unaltered by sevoflurane general anesthesia

**DOI:** 10.1097/PR9.0000000000000936

**Published:** 2021-06-03

**Authors:** Eunice Y. Hahm, Shubham Chamadia, Joseph J. Locascio, Juan C. Pedemonte, Jacob Gitlin, Jennifer Mekonnen, Reine Ibala, Breanna R. Ethridge, Katia M. Colon, Jason Qu, Oluwaseun Akeju

**Affiliations:** a Department of Anesthesia, Critical Care and Pain Medicine, Massachusetts General Hospital, Boston, MA, USA; b Department of Neurology, Massachusetts General Hospital, Boston, MA, USA; c División de Anestesiología, Escuela de Medicina, Pontificia Universidad Católica de Chile, Santiago, Chile; d Henry and Allison McCance Center for Brain Health, Massachusetts General Hospital, Boston, MA, USA

**Keywords:** Ketamine, Dissociation, Pain, Analgesia, Sevoflurane

## Abstract

**Introduction:**

Ketamine, an anesthetic adjunct, is routinely administered as part of a balanced general anesthetic technique. We recently showed that the acute analgesic and dissociation properties of ketamine are separable to suggest that distinct neural circuits underlie these states.

**Objective:**

We aimed to study whether this finding is robust to the substantial neural circuit alterations associated with general anesthesia.

**Methods:**

We conducted a single-site, open-label, randomized controlled, cross-over study of sevoflurane and sevoflurane-plus-ketamine (SK) general anesthesia in healthy subjects (n = 12). Before and after general anesthesia, we assessed precalibrated cuff pain intensity and nociceptive pain quality as well as dissociation using the Clinician-Administered Dissociative States Scale (CADSS). For statistical inference, we ran a variation of backward elimination repeated-measures analysis of covariance. Models with CADSS as a covariate term were used to assess whether dissociation mediated the effect of ketamine on pain intensity and quality.

**Results:**

Sevoflurane-plus-ketamine general anesthesia was associated with a significant (*P* = 0.0002) pain intensity decline of 3 (SE, 0.44). There was an order effect for dissociation such that SK was associated with a significant (*P* = 0.0043) CADSS increase of 17.8 (3.2) when the SK treatment came first. When the pain intensity model was reanalyzed with CADSS as an additional covariate, the effect of CADSS was not significant. These results were also conserved for pain quality.

**Conclusions:**

Our findings suggest that the analgesic and dissociation properties of ketamine remain separable despite general anesthesia. Thus, ketamine may be used as a probe to advance our knowledge of dissociation independent pain circuits.

## Abstract 2

Ketamine-induced dissociation and analgesia are independent and robust to general anesthesia neural circuit alterations, suggesting that ketamine can be refined into a targeted pain therapeutic.

## 1. Introduction

Ketamine is associated with acute analgesia, antihyperalgesia, modulation of opioid-mediated analgesia, and dissociation.^3,13,19,25^ The dissociative properties of ketamine, such as detachment feelings, have largely limited its widespread use as an analgesic medication. We recently showed that the acute analgesic and dissociation properties of ketamine are separable to suggest that ketamine or its metabolites modulate distinct neural circuits to produce dissociation and analgesia.^10^

General anesthetic drugs significantly modulate neural circuits to produce unconsciousness, amnesia, antinociception, and immobility.^1^ Ketamine is routinely administered as an anesthetic adjunct as part of a balanced general anesthetic technique.^2,7,12,23^ However, it is not clear whether the separable analgesic and dissociation properties of ketamine are robust to the substantial alterations of neural circuit activity associated with general anesthesia.^1^ Therefore, we analyzed data obtained from a single-site, open-label, randomized, controlled, cross-over study of sevoflurane (S) general anesthesia and sevoflurane-plus-ketamine (SK) general anesthesia (n = 12).

## 2. Methods

**Figure 1.**
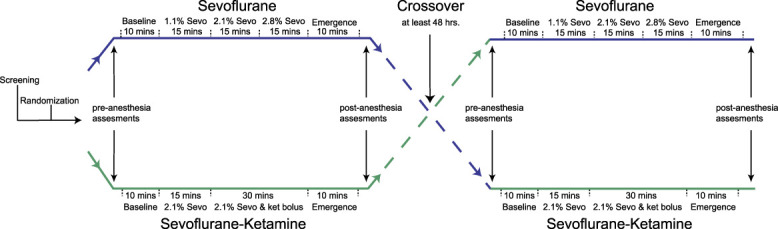
Schematic of the study protocol. We conducted the study using a single-site, randomized, controlled, cross-over design (n = 12). For each subject, during the sevoflurane-ketamine visit, we administered an intravenous bolus of ketamine (0.75 mg/kg). Each subject underwent both the sevoflurane and the sevoflurane-plus-ketamine treatment conditions (within-subject factor), but subjects were randomized to the condition of which treatment came first (between-subject factor). We assessed pain intensity using the PROMIS questionnaire and dissociative symptoms using the Clinician-Administered Dissociative States Scale (CADSS). PROMIS, Patient-Reported Outcomes Measures Information System.

The Partners Institutional Review Board approved this human research study (2014P000111) registered on www.ClinicalTrials.gov (NCT03503578). Details of our subject recruitment and study design have previously been reported.^7^ This study reports the secondary analysis (pain and dissociation measures), distinct from our primary analysis of the electroencephalogram dynamics associated with sevoflurane-induced general anesthesia.^7^ In brief, we induced and allowed recovery from S general anesthesia and SK general anesthesia in 12 healthy subjects using a cross-over study design (Fig. 1). During the S visit, after 10 minutes of baseline (awake) state, we increased the end-tidal sevoflurane concentration in a stepwise fashion to subanesthetic (1.1%), general anesthetic (2.1%), and deep-general anesthetic (2.8%) states. Each anesthetic state was maintained for 15 minutes. During the SK visit, after 10 minutes of the baseline state, we increased the sevoflurane end-tidal concentration to a general anesthetic (2.1%) state and maintained the anesthetic concentration for 45 minutes. We also administered an intravenous bolus of ketamine (0.75 mg/kg) after achieving 15 minutes of steady-state sevoflurane concentration. Finally, we assessed 10 minutes of emergence state during each visit. Study visits were separated by at least 48 hours.

### 2.1. Pain and dissociation measures

All subjects underwent a baseline pain stimuli calibration using a validated pneumatic cuff pain device (Hokanson Rapid Cuff Inflator)^20,21^ that delivered the pain stimulus to the gastrocnemius area of the lower leg. This cuff pain device is a computer-controlled air compressor that inflates and maintains the pressure at the desired level. We assessed pain intensity and quality using the standard Patient-Reported Outcomes Measures Information System (PROMIS) Pain Intensity 1A and PROMIS Nociceptive Pain Quality 5a. We also assessed for dissociation using the Clinician-Administered Dissociative States Scale (CADSS). The CADSS is a standardized clinical measure of perceptual, behavioral, and attentional alterations during dissociative experiences.^5^ Both pain and dissociation assessments were conducted before drug administration and after recovery.

### 2.2. Statistical methods

We ran a variation of a mixed-effects repeated-measures analysis of covariance including the between-subject factor of the order of treatment (S-SK and SK-S) fully crossed with the within-subject factor of the type of anesthesia (S and SK), which was further fully crossed with the within-subject factor of the assessment period (before and after). An unstructured covariance matrix for the repeated-measures factors was used. The between-subject covariates of age and gender were also included. An initial model including all the above-mentioned terms and all 2-way and 3-way interactions among the assessment, anesthesia, and order factors, was run, as well as a limited backward elimination model excluding the least significant term (*P* > 0.05) sequentially until only significant terms remained or nonsignificant terms subsumed within significant higher-order terms. The above-mentioned model was run separately for each dependent variable: PROMIS Pain Intensity, PROMIS Nociceptive Pain, and CADSS. Also, further models were run in which each pain measure was the dependent variable but with an additional condition-varying covariate assessing dissociation (CADSS) to assess the possible role of dissociation in mediating effects of ketamine on pain. Tukey–Kramer adjusted post-hoc tests of mean differences were run as pertinent follow-up tests for significant omnibus analysis of variance effects. Residuals from all fit models were examined for conformance to the assumptions of normality. Statistical analyses were performed with SAS version 9.4 (Cary, NC).

## 3. Results

**Figure 2.**
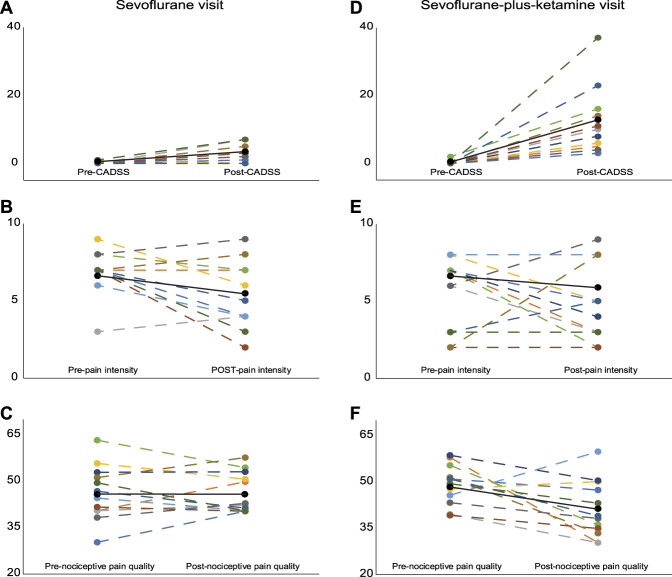
“Spaghetti” plots for analgesic and dissociative effects. During the sevoflurane visit, we conducted preanesthetic and postanesthetic assessments of (A) dissociation using the CADSS score, (B) pain intensity using PROMIS scores, and (C) nociceptive pain. Similarly, during the sevoflurane-plus-ketamine visit, we conducted preanesthetic and postanesthetic assessment of (D) dissociation using the CADSS score, (E) pain intensity using PROMIS scores, and (F) nociceptive pain. Subject data are color-coded, with each dashed line connecting scores for the same subject. Solid black line connects the means at the pretest assessment to the post test assessment. Data for the different orders of treatment (S-KS and KS-S) are pooled in this figure. CADSS, Clinician-Administered Dissociative States Scale; PROMIS, Patient-Reported Outcomes Measures Information System.

Results from PROMIS and CADSS assessments are summarized in Figure 2. Residuals from all models below conformed reasonably to the normality assumption.

### 3.1. Pain intensity and nociceptive pain models

The final pain intensity backward elimination model included significant effects for age (*P* = 0.0515; older with less pain) and for the interaction of assessment × anesthesia (*P* = 0.052; larger decrease after SK visit). Post-hoc analysis of this interaction term showed that the SK-visit pain mean intensity decline of 3 (SE, 0.44) was significant (*P* = 0.0002, Tukey–Kramer; *P* < 0.0001, uncorrected), whereas a decline of 1.33 (0.62) for S visit was not significant. The retained predictors for the model accounted for 43% of the variance in pain intensity. These data are summarized in Table 1.

**Table 1:** Pain intensity final backward elimination model analysis.

Effect	Assessment	Anesthesia	Adjusted means	SE
Assessment × anesthesia	Pre	S	6.9	0.4
Assessment × anesthesia	Pre	SK	6.8	0.3
Assessment × anesthesia	Post	S	5.6	0.7
Assessment × anesthesia	Post	SK	3.8	0.5

SK, sevoflurane-plus-ketamine.

The final nociceptive pain model also revealed a significant assessment × anesthesia interaction (*P* = 0.049; reflecting again a larger decrease after SK visit). Post-hoc analysis of this interaction term showed that the SK-visit nociceptive pain mean decline of 8.1 (2.7) was significant (*P* = 0.0523, Tukey–Kramer; *P* = 0.0125, uncorrected), whereas the analogous decline for S visit of 0.14 (2.29) was negligible and nonsignificant. The retained predictors for the model accounted for 13% of the variance in nociceptive pain. These data are summarized in Table 2.

**Table 2:** Nociceptive pain quality final backward elimination model analysis.

Effect	Assessment	Anesthesia	Adjusted means	SE
Anesthesia × assessment	Pre	S	46.3	2.1
Anesthesia × assessment	Pre	SK	49.2	2.5
Anesthesia × assessment	Post	S	46.1	2.0
Anesthesia × assessment	Post	SK	41.1	2.5

SK, sevoflurane-plus-ketamine.

### 3.2. Clinician-Administered Dissociative States Scale model

The final model for the CADSS revealed a significant 3-way interaction of order x anesthesia x assessment (*P* = 0.027; larger increase after SK visit, especially when SK visit came first). Changes in the CADSS adjusted means from preanesthesia to postanesthesia for the S visit did not meet our statistical significance threshold. The increase in pre–CADSS-adjusted to post–CADSS-adjusted means for the SK visit under the S-SK randomization order was 7.14 (SE, 2.8) and not significant (*P* = 0.2574, Tukey–Kramer; *P* = 0.0264, uncorrected). For the same comparison under the SK-S randomization order, the increase was 17.8 (SE, 3.2) and significant (*P* = 0.0043, Tukey–Kramer; *P* = 0.0003, uncorrected). Approximately 62% of the variance in the CADSS was accounted for by the model. These data are summarized in Table 3.

**Table 3:** Clinician-Administered Dissociative States Scale final backward elimination model analysis.

Effect	Order	Anesthesia	Assessment	Adjusted means	SE
Order × anesthesia × assessment	Sev	S	Pre	0.3	0.2
Order × anesthesia × assessment	Sev	S	Post	2.7	1.4
Order × anesthesia × assessment	Sev	SK	Pre	0.3	0.3
Order × anesthesia × assessment	Sev	SK	Post	7.4	2.8
Order × anesthesia × assessment	Ket	S	Pre	0.2	0.2
Order × anesthesia × assessment	Ket	S	Post	3.4	1.7
Order × anesthesia × assessment	Ket	SK	Pre	−21E-16	0.3
Order × anesthesia × assessment	Ket	SK	Post	17.8	3.3

Ket, sevoflurane-plus-ketamine visit first; Pre, preanesthetic assessment; Post, postanesthetic assessment; S, sevoflurane general anesthesia; SE, standard error; Sev, sevoflurane-visit first; SK, sevoflurane-plus-ketamine general anesthesia.

### 3.3. Pain intensity and nociceptive pain models, covarying the Clinician-Administered Dissociative States Scale

Finally, we assessed whether there were any effects for pain intensity and nociceptive pain covarying for the CADSS, ie, running the same model above-mentioned for pain intensity and nociceptive pain but including the CADSS as a term in the initial model. The CADSS term was removed as nonsignificant in both models, leaving the same final models reported above. Before being removed from the pain intensity model, the nonsignificant (*P* = 0.25) adjusted coefficient for the CADSS was estimated as −0.05 (with a 95% confidence interval: −0.14 to 0.04), indicating that at most, the pain intensity measure decreased by 0.14 units for a 1 unit increment in the CADSS. The corresponding estimate for the nonsignificant (*P* = 0.47) CADSS effects for nociceptive pain was −0.16 (confidence interval: −0.61 to 0.29).

## 4. Discussion

In this investigation, we studied the effect of sevoflurane general anesthesia and sevoflurane ketamine general anesthesia on dissociation and analgesia. Our major finding was that the acute analgesic and dissociative properties of ketamine are separable and robust to the neural circuit alterations associated with general anesthesia.

Vesuna et al. recently found that hyperpolarization-activated cyclic nucleoside-gated 1 (HCN1) channels in the retrosplenial cortex are needed for dissociation-like behavior in mice.^24^ Inhibition of glutaminergic and excitatory long-range inputs to the retrosplenial cortex, which is likely modulated by sevoflurane general anesthesia,^4,6,9,22^ may comprise this dissociation-like behavior circuit.^24^ Our findings suggest that this dissociation circuit and the circuit underlying ketamine's analgesic properties are not entwined by sevoflurane. In addition to HCN1 channels, we note that ketamine also interacts with N-methyl-d-aspartate, opioid, monoaminergic, cholinergic, nicotinic, and muscarinic receptors.^18,26^ However, it is unlikely that the analgesic properties of ketamine are secondary to opioid receptor agonist activity.^16,17^

Our finding is expected to motivate studies to define how ketamine acts in neural circuits to produce analgesia.^13^ These studies may also lend insights into desirable antidepressant and antisuicidality properties of ketamine.^14,15^ The strengths of our study include structured pain and dissociation assessments. However, a key limitation was that our washout period of 48 hours was based on pharmacokinetics, as the putative longer-term duration of general anesthesia–induced neural circuit alterations is unclear. Thus, future studies are necessary to lend insights into the duration of neural circuit alterations associated with anesthetic drugs. These insights may benefit our understanding of the mechanism underlying the order effect we found for ketamine-induced dissociation (ie, there was an order effect for ketamine-induced dissociation scores). In addition, we did not measure the blood levels of metabolites that might influence the analgesic effect of administered anesthetic drugs.

We conclude that ketamine-induced dissociation and analgesia are independent, suggesting that ketamine can be refined into a more targeted pain therapeutic.^8,11^ We also conclude that ketamine may be used as a probe to advance our knowledge of dissociation independent pain circuits. We found that dissociation did not entirely mediate the relation of analgesics to pain reduction although it may partly do so, and further replication of these effects would be desirable.

## Disclosures

O. Akeju has received speaker's honoraria from Masimo Corporation and is listed as an inventor on pending patents on EEG monitoring and oral dexmedetomidine assigned to Massachusetts General Hospital. The remaining authors have no conflicts of interest to declare.

NIH NIA RO1AG053582 to O. Akeju; and, Innovation funds from the Department of Anesthesia, Critical Care, and Pain Medicine, Massachusetts General Hospital to O. Akeju; funds from División de Anestesiología, Escuela de Medicina, Pontificia Universidad Católica de Chile to J.C. Pedemonte.
